# Regular Breakfast Consumption and Type 2 Diabetes Risk Markers in 9- to 10-Year-Old Children in the Child Heart and Health Study in England (CHASE): A Cross-Sectional Analysis

**DOI:** 10.1371/journal.pmed.1001703

**Published:** 2014-09-02

**Authors:** Angela S. Donin, Claire M. Nightingale, Chris G. Owen, Alicja R. Rudnicka, Michael R. Perkin, Susan A. Jebb, Alison M. Stephen, Naveed Sattar, Derek G. Cook, Peter H. Whincup

**Affiliations:** 1 Population Health Research Institute, Division of Population Health Sciences and Education, St George's University of London, London, United Kingdom; 2 Nuffield Department of Primary Care Health Sciences, University of Oxford, Oxford, United Kingdom; 3 Medical Research Council Human Nutrition Research, Cambridge, United Kingdom; 4 Institute of Cardiovascular and Medical Sciences, University of Glasgow School of Medicine, Glasgow, United Kingdom; Simon Fraser University, Canada

## Abstract

**Background:**

Regular breakfast consumption may protect against type 2 diabetes risk in adults but little is known about its influence on type 2 diabetes risk markers in children. We investigated the associations between breakfast consumption (frequency and content) and risk markers for type 2 diabetes (particularly insulin resistance and glycaemia) and cardiovascular disease in children.

**Methods and Findings:**

We conducted a cross-sectional study of 4,116 UK primary school children aged 9–10 years. Participants provided information on breakfast frequency, had measurements of body composition, and gave fasting blood samples for measurements of blood lipids, insulin, glucose, and glycated haemoglobin (HbA1c). A subgroup of 2,004 children also completed a 24-hour dietary recall. Among 4,116 children studied, 3,056 (74%) ate breakfast daily, 450 (11%) most days, 372 (9%) some days, and 238 (6%) not usually. Graded associations between breakfast frequency and risk markers were observed; children who reported not usually having breakfast had higher fasting insulin (percent difference 26.4%, 95% CI 16.6%–37.0%), insulin resistance (percent difference 26.7%, 95% CI 17.0%–37.2%), HbA1c (percent difference 1.2%, 95% CI 0.4%–2.0%), glucose (percent difference 1.0%, 95% CI 0.0%–2.0%), and urate (percent difference 6%, 95% CI 3%–10%) than those who reported having breakfast daily; these differences were little affected by adjustment for adiposity, socioeconomic status, and physical activity levels. When the higher levels of triglyceride, systolic blood pressure, and C-reactive protein for those who usually did not eat breakfast relative to those who ate breakfast daily were adjusted for adiposity, the differences were no longer significant. Children eating a high fibre cereal breakfast had lower insulin resistance than those eating other breakfast types (*p* for heterogeneity <0.01). Differences in nutrient intakes between breakfast frequency groups did not account for the differences in type 2 diabetes markers.

**Conclusions:**

Children who ate breakfast daily, particularly a high fibre cereal breakfast, had a more favourable type 2 diabetes risk profile. Trials are needed to quantify the protective effect of breakfast on emerging type 2 diabetes risk.

*Please see later in the article for the Editors' Summary*

## Abstract 2

Angela Donin and colleagues evaluated the association between breakfast consumption and composition and risk markers for diabetes and cardiovascular disease in 9- and 10-year-olds.

*Please see later in the article for the Editors' Summary*

## Editors' Summary

**Background:**

Worldwide, more than 380 million people have diabetes, a disorder that is characterized by high levels of glucose (sugar) in the blood. Blood sugar levels are usually controlled by insulin, a hormone released by the pancreas after meals (digestion of food produces glucose). In people with type 2 diabetes (the commonest type of diabetes) blood sugar control fails because the fat and muscle cells that normally respond to insulin become insulin resistant. Type 2 diabetes can often be controlled initially with diet and exercise and with drugs such as metformin and sulfonylureas. However, many patients eventually need insulin injections to control their blood sugar levels. Long-term complications of diabetes, which include an increased risk of heart disease and stroke (cardiovascular disease), reduce the life expectancy of people with diabetes by about 10 years compared to people without diabetes. Risk factors for the condition include being over 40 years old and being overweight or obese.

**Why Was This Study Done?:**

Experts predict that by 2035 nearly 600 million people will have diabetes so better strategies to prevent diabetes are urgently needed. Eating breakfast regularly—particularly a high fiber, cereal-based breakfast—has been associated with a reduced risk of type 2 diabetes (and a reduced risk of being overweight or obese) in adults. However, little is known about whether breakfast eating habits affect markers of type 2 diabetes risk in children. In this cross-sectional study (an observational investigation that studies a group of individuals at a single time point), the researchers examine the associations between breakfast consumption (both frequency and content) and risk markers for type 2 diabetes, particularly insulin resistance and glycemia (the presence of sugar in the blood), in an ethnically mixed population of children; insulin resistance and glycemia measurements in children provide important information about diabetes development later in life.

**What Did the Researchers Do and Find?:**

The researchers invited 9–10 year old children attending 200 schools in London, Birmingham, and Leicester to participate in the Child Heart and Health Study in England (CHASE), a study examining risk factors for cardiovascular disease and type 2 diabetes in children of South Asian, black African-Caribbean, and white European origin. The researchers measured the body composition of the study participants and the levels of insulin, glucose, and other markers of diabetes risk in fasting blood samples (blood taken from the children 8–10 hours after their last meal or drink). All the participants (4,116 children) reported how often they ate breakfast; 2,004 children also completed a 24-hour dietary recall questionnaire. Seventy-four percent of the children reported that they ate breakfast every day, 11% and 9% reported that they ate breakfast most days and some days, respectively, whereas 6% reported that they rarely ate breakfast. Children who ate breakfast infrequently had higher fasting insulin levels and higher insulin resistance than children who ate breakfast every day. Moreover, the children who ate a high fiber, cereal-based breakfast had lower insulin resistance than children who ate other types of breakfast such as low fiber or toast-based breakfasts.

**What Do These Findings Mean?:**

These findings indicate that children who ate breakfast every day, particularly those who ate a high fiber breakfast, had lower levels of risk markers for type 2 diabetes than children who rarely ate breakfast. Importantly, the association between eating breakfast and having a favorable type 2 diabetes risk profile remained after allowing for differences in socioeconomic status, physical activity levels, and amount of body fat (adiposity); in observational studies, it is important to allow for the possibility that individuals who share a measured characteristic and a health outcome also share another characteristic (a confounder) that is actually responsible for the outcome. Although trials are needed to establish whether altering the breakfast habits of children can alter their risk of developing type 2 diabetes, these findings are encouraging. Specifically, they suggest that if all the children in England who do not eat breakfast daily could be encouraged to do so, it might reduce population-wide fasting insulin levels by about 4%. Moreover, encouraging children to eat a high fiber breakfast instead of a low fiber breakfast might reduce population-wide fasting insulin levels by 11%–12%. Thus, persuading children to eat a high fiber breakfast regularly could be an important component in diabetes preventative strategies in England and potentially worldwide.

**Additional Information:**

Please access these websites via the online version of this summary at http://dx.doi.org/10.1371/journal.pmed.1001703.

- The US National Diabetes Information Clearinghouse provides information about diabetes for patients, health-care professionals, and the general public, including detailed information on diabetes prevention (in English and Spanish)
- The UK National Health Service Choices website provides information for patients and carers about type 2 diabetes and about living with diabetes; it also provides people's stories about diabetes; Change4Life, a UK campaign that provides tips for healthy living, has a webpage about the importance of a healthy breakfast
- The charity Diabetes UK provides detailed information for patients and carers in several languages, including information on healthy lifestyles for people with diabetes
- The UK-based non-profit organization Healthtalkonline has interviews with people about their experiences of diabetes
- MedlinePlus provides links to further resources and advice about diabetes and diabetes prevention (in English and Spanish)
- Kidshealth, a US-based not-for-profit organization provides information for parents about the importance of breakfast and information for children
- More information about the Child Heart and Health Study in England (CHASE) is available

## Introduction

The high prevalence of type 2 diabetes both globally and in the UK, which affects increasingly younger individuals [1], presents a major public health challenge [2]. Diet and eating patterns appear to play an important role in the aetiology of type 2 diabetes, though the importance of specific dietary components remains unresolved [2]. Breakfast is an important meal, providing appreciable proportions of daily energy, macronutrient, and micronutrient intakes [3]. In adults, skipping breakfast has been associated with higher risk of overweight and obesity [4],[5] and with a higher risk of type 2 diabetes [6],[7]. In young adults, skipping breakfast has been associated with an increased risk of the metabolic syndrome [8]. Breakfast content may also be important; studies in the US have shown that the consumption of breakfast cereal is associated with a more favourable risk factor profile for type 2 diabetes and cardiovascular disease [9].

Previous studies in children have shown consistent associations between skipping breakfast and higher body mass and obesity prevalence [10]. However, the influence of breakfast frequency on precursors of type 2 diabetes and cardiovascular disease in children have not been reported to date. Moreover, among children who do eat breakfast, the influence of breakfast content has been little studied. In addition, although studies have shown that children who report skipping breakfast have poorer diet quality, with lower energy intakes, higher energy density, saturated fat, and lower vitamin and mineral intakes [10], the contribution of these nutritional differences to differences in adiposity or diabetes risk remains uncertain. These questions are particularly important because recent evidence suggests that the prevalence of breakfast consumption has declined both in adults and children [11],[12]. In the UK, these issues may be particularly relevant for children from ethnic minority groups (especially children of South Asian and black African-Caribbean origin) who are at increased risk of both type 2 diabetes and obesity [13] and may be less likely to eat breakfast every day [14].

We investigated the associations between breakfast consumption (both frequency and breakfast content) and risk markers for type 2 diabetes and cardiovascular disease in a large multi-ethnic population of children in order to test our main research hypothesis that both breakfast frequency and composition would be associated with type 2 diabetes risk markers in childhood, and particularly with insulin resistance and glycaemia, which are strongly related to the development of insulin resistance, hyperglycaemia, the metabolic syndrome, and type 2 diabetes in adult life [15]. In a subset of the study population with detailed dietary information, we also examined differences in dietary energy and nutrient intakes between children who regularly eat breakfast and those who do not, and their contribution to observed differences in risk markers for type 2 diabetes and cardiovascular disease.

## Methods

### Ethics Statement

Ethical approval was provided by the Multicentre Research Ethics Committee Wales.

This investigation was based on the Child Heart And health Study in England (CHASE) [16], which examined risk markers for type 2 diabetes and cardiovascular disease risk and their determinants in a multi-ethnic population of children aged 9–10 years. Children of predominantly South Asian, black African-Caribbean, and white European origin were invited to take part, drawn from a stratified random sample of 200 primary schools in London, Birmingham, and Leicester. Numbers of participants from different ethnic groups were balanced in order to maximize power for ethnic group comparisons.

The school response rate was 70%; all schools that declined to participate were replaced by a similar school from the sampling frame in the same borough. Data were collected between October 2004 and February 2007. All year 5 children in the selected schools were invited to take part. Parents or guardians provided informed written consent. All participating children completed questionnaires (including a question on breakfast frequency), had physical measurements, and provided a fasting blood sample. In the last 85 schools (visited between February 2006 and February 2007 and similar in size, borough location, and ethnic composition to the preceding 115 schools), detailed dietary information was collected with a 24-hour recall assessment and objective physical activity measurements were made using Actigraph GT1M movement sensors (Actigraph) over a 7-day period, as described in detail elsewhere [17]. Information on breakfast frequency was therefore available for all participants and information on breakfast content (and other aspects of nutrient intakes) for a subset of children.

### Assessments Made in All Children

All participating children were asked to complete a questionnaire that included a question on whether they usually ate breakfast in the morning with the following four options; every day, most days, some days, or not usually. Three trained observers made measurements of height, weight, and bioelectrical impedance, measured with a Bodystat 1500 body composition analyser (Bodystat Ltd). Fat free mass was obtained from bioelectrical impedance using validated equations derived specifically for UK children of this age group, which were sex and ethnic group specific [18]. Fat mass was obtained by subtracting fat free mass from total body weight and was presented as a height-standardized index (fat mass [kg]/height [m]^5^) [19]. Fat mass index from bioelectrical impedance was used as the principal marker of body fat as it provides valid measurements of body fat in this multi-ethnic population, in contrast with body mass index, which yields biased results [19]. Skinfold thickness was measured at the biceps, triceps, subscapular, and suprailiac locations. Seated blood pressure was measured twice in the right arm after 5 min of rest using an Omron 907 blood pressure recorder, with an appropriately sized cuff. Children provided blood samples after an overnight fast for the measurement of all blood markers including total and high density lipoprotein (HDL) cholesterol and triglycerides; low density lipoprotein (LDL) cholesterol was obtained using the Fredrickson-Friedewald equation [20]. Serum insulin was measured using an ELISA method [21], plasma glucose using the glucose oxidase method. Glycated haemoglobin (HbA1c) was measured in whole blood by ion exchange high performance liquid chromatography. The homeostasis model assessment (HOMA) equations were used to provide an estimate of insulin resistance [22], and C-reactive protein was assayed by ultra-sensitive nephelometry (Dade Behring). Serum urate was assayed using an enzymatic method [23]. Ethnicity of the child was categorised using self-defined ethnicity for both parents or by using parental information on the ethnicity of the child. In a small number of participants for whom this information was not available (1%), child defined place of origin of parents and grandparents was used cross-checked with the observer defined ethnic appearance of the child. Children were broadly classified into four main ethnic groups (“white European,” “black African-Caribbean,” “South Asian,” “other”) with a more detailed classification into ten ethnic subgroups (white European, black African, black Caribbean, black other, Indian, Pakistani, Bangladeshi, South Asian other, Asian other, other) for ethnicity adjustments in analysis. The ethnic sub-groups “Indian,” “Pakistani,” and “Bangladeshi” were restricted to children whose parents both originated in the same country; “black African” and “black Caribbean” groups were restricted to those who originated in the same region. Parents and children provided information on parental occupation, which was then coded using the National Statistics-Socioeconomic Classification (NS-SEC) [24], resulting in the following classifications: managerial/professional, intermediate, routine/manual, and economically inactive (refers to people currently unemployed, whether or not they are seeking work).

### Detailed Dietary Assessment and Physical Activity Measurement (Subset of Children)

Children in the last 85 schools (February 2006–February 2007) were interviewed by a research nutritionist and a single, structured 24-hour recall of foods eaten the previous day was conducted, in accordance with the recommendations of the Nordic Cooperation of Dietary Researchers [25] and included key elements of the United States Department of Agriculture (USDA) multiple pass method [26]. Memory cues were used to aid recall, such as orientating the child on details of the previous day, and checking for any forgotten snacks or drinks that the child may have had through the day. Photographs of common foods were used to help the child estimate portion sizes. Nutrient intakes were then calculated by the Medical Research Council Human Nutrition Research centre (MRC-HNR) using an in-house food composition database. Energy density was calculated by dividing the reported total energy intake from food (kJ) by the total weight of food reported (g), excluding all drinks [27]. The details of the breakfast meal and its component nutrients were specifically identified to allow characterisation of breakfast contents into five categories; high fibre cereal (≥3 g/40 g portion including oat-based breakfasts), low fibre cereal (<3 g/40 g portion), bread-based breakfast only, biscuit-based breakfast only, and other (which included eggs, fruit, and yogurts). Information collected from the children lacked sufficient detail to be able to consistently code the bread-based breakfast as high fibre or low fibre, however the majority of children (62%) reported having white bread at home. Children were also asked to wear Actigraph GT1M movement sensors (Actigraph) over a 7-day period, from which an objective measure of physical activity (counts per minute) was derived, as described in detail elsewhere [17].

### Statistical Methods

Statistical analyses were carried out using STATA/SE software (STATA/SE 12 for Windows, StataCorp LP). Multilevel linear regression models were used to provide adjusted means (adjusted to the average level of each variable in the model, so that the values are close to the observed data) and to quantify the associations between breakfast frequency, risk markers, and dietary intake, using XTMIXED and LINCOM commands. All analyses were adjusted for sex, age in quartiles, ethnicity (in ten ethnic subgroups), day of week and month as fixed effects; school was fitted as a random effect to allow for the clustering of children within schools. No adjustments were made for multiple comparisons as a strong a priori hypothesis that both breakfast frequency and content will be associated with insulin resistance and glycaemia was to be tested.

## Results

Among 8,641 pupils invited, 5,887 (68%) took part in the study. Of these, 4,841 children provided fasting blood samples and had physical measurements (82% of the sample); 4,116 children (2,164 girls [53%] and 1,952 boys) also provided information on usual breakfast frequency and were therefore included in the present analyses. Table S1 provides data on socio-demographic characteristics and key risk markers for study participants who were included or excluded (because of incompleteness of data) from main analyses. Subjects excluded were slightly more likely to be boys, to be of black African-Caribbean origin, and to have economically inactive parents, though their risk markers for type 2 diabetes and cardiovascular disease did not differ appreciably. The mean age of participants was 10.0 years (95% reference range 9.3 to 10.6 years). There were similar numbers of children of white European, black African Caribbean, South Asian, and other ethnic groups (979, 1,056, 1,118, and 963, respectively). In the dietary survey in the final 85 schools visited, 2,004 children completed 24-hour recalls and also provided physical measurements and fasting blood samples. There was close agreement between reported breakfast frequency and the presence/absence of a breakfast meal on the 24-hour call among children with data from both sources. Among children who reported eating breakfast daily, most days, some days, and not usually, the proportions with a breakfast meal documented in their 24-hour recall were 94%, 85%, 61%, and 30%, respectively. Table 1 presents the socio-demographic characteristics, type 2 diabetes and cardiovascular risk markers, physical activity, and diet for all study participants by reported breakfast frequency In total, 26% of children reported not having breakfast every day; this included 11% of children who reported eating breakfast most days, 9% on some days, and 6% not usually. The proportions of children not having breakfast were similar in boys and girls but differed between ethnic and socioeconomic groups. Black African Caribbean children most frequently reported not having breakfast every day (30%) compared to 25% of South Asian children and 22% of white European children (*p* = 0.001). Children from the lowest socioeconomic category were more likely not to have breakfast every day (36%) compared with those in the managerial group (20%) (*p*<0.0001).

**Table 1:** Socio-demographic characteristics, type 2 diabetes and cardiovascular risk markers, diet and physical activity for all study participants by breakfast frequency.

Characteristics	Breakfast frequency reported as								*p*-Value
	Every day		Most days		Some days		Not usually		
**All children (n = 4,116)**									
All children (n, %)	3,056	74	450	11	372	9	238	6	
Boys (n, %)	1,482	76	199	10	168	9	103	5	
Girls (n, %)	1,574	73	251	12	204	9	135	6	0.13
**Ethnicity:**									
White European (n, %)	769	79	100	10	67	7	43	4	
Black African Caribbean (n, %)	735	70	125	12	113	10	83	8	
South Asian (n, %)	834	75	119	10	99	9	66	6	
Other (n, %)	718	74	106	11	93	10	46	5	0.001
**Parental occupation:**									
Managerial (n, %)	923	80	123	11	62	5	52	4	
Intermediate (n, %)	808	75	111	10	93	9	64	6	
Routine and manual (n, %)l	923	74	121	10	129	10	71	6	
Economically inactive (n, %)	260	64	61	15	56	14	29	7	<0.0001
Age (years)	9.95	0.4	9.98	0.4	9.99	0.41	9.97	0.39	0.13
Fat mass index (kg/m^5^)^a^	2.00	1.47	2.11	1.49	2.15	1.53	2.26	1.52	<0.0001
Sum of skinfolds (mm)^a^	39.74	1.63	43.92	1.64	44.09	1.66	49.19	1.68	<0.0001
Leptin (ng/mL)^a^	8.64	2.59	10.00	2.49	10.45	2.63	12.31	2.63	<0.0001
Insulin (mmol/L)^a^	6.93	1.90	8.00	1.88	8.45	1.89	9.07	1.81	<0.0001
Insulin resistance (HOMA)^a^	0.87	1.89	1.01	1.87	1.07	1.88	1.14	1.78	<0.0001
HbA1c (%)^a^	5.24	1.07	5.24	1.07	5.27	1.07	5.31	1.06	0.007
Glucose (mmol/L)^a^	4.49	1.08	4.55	1.08	4.60	1.08	4.54	1.09	<0.0001
C-reactive protein (mg/L)^a^	0.48	3.68	0.56	3.60	0.55	4.07	0.69	4.26	<0.0001
Urate (mmol/L)^a^	0.22	1.29	0.22	1.29	0.22	1.27	0.23	1.25	0.004
Triglycerides (mmol/L)^a^	0.80	1.49	0.81	1.48	0.81	1.49	0.83	1.47	0.50
Total cholesterol (mmol/L)	4.56	0.77	4.53	0.75	4.52	0.76	4.59	0.8	0.50
LDL-cholesterol (mmol/L)	2.68	0.67	2.66	0.61	2.67	0.63	2.72	0.64	0.74
HDL-cholesterol (mmol/L)	1.52	0.31	1.49	0.34	1.48	0.28	1.49	0.32	0.004
Systolic BP (mmHg)	104.4	10.6	105.3	10.2	105.6	10.2	105.2	10	0.07
Diastolic BP (mmHg)	62.7	9.4	62.8	9.4	63.3	9.2	63.2	9	0.69
Physical activity (counts per min)*	480.6	107.7	495.7	105.4	472.7	103.7	476	123	0.39
**Dietary intake (n = 1,899)**									
Energy (kcals)	1,863	494	1,826	480	1792	512	1,578	432	0.0001
Energy density	7.0	1.6	7.1	1.5	7.3	1.7	7.4	2.1	0.01
Fat % energy	34.1	6.4	34.5	6.2	35.4	6.5	36.2	6.4	0.02
Saturated fat % energy	12.7	3.4	12.5	3.6	12.6	3.6	12.8	3.4	0.89
Monounsaturated fat % energy	11.3	2.8	11.4	2.8	11.8	2.7	12.5	3.0	0.002
Polyunsaturated fat % energy	6.5	3.1	6.9	2.8	6.9	3.1	6.8	2.8	0.14
Carbohydrate % energy	52.2	6.7	51.9	6.8	51.1	7.0	49.4	7.3	0.005
Sugar % energy	22.5	6.9	22.4	7.0	22.2	7.1	20.1	7.8	0.21
Starch % energy	29.2	6.1	29.1	6.2	28.3	6.0	28.0	7.6	0.21
NSP (g)	12.0	4.8	11.5	4.2	11.3	4.8	10.6	5.0	0.03
Protein % energy	13.3	3.2	13.3	3.5	13.3	3.6	14.2	4.3	0.20

Values shown are mean, SD unless otherwise indicated. *p*-Values test for unordered differences between breakfast groups and are derived from chi^2^ tests (for socio-demographic variables) and ANOVA tests for all other continuous variables. NSP, non-starch polysaccharides.

*Based on 1,581 subjects.

a Variable is log transformed.

Geometric means and geometric standard deviations are presented for log transformed variables. See Table S7 for raw data for these variables.

### Breakfast Frequency and Risk Markers for Type 2 Diabetes and Cardiovascular Disease

Table 2 presents the adjusted mean values for the risk markers by reported breakfast frequency, in analyses adjusted for age, sex, month, ethnicity, and school (random effect), with formal tests for trend across the groups. Insulin resistance, HbA1c, glucose, triglyceride, C-reactive protein, urate, systolic blood pressure, fat mass index, and sum of skinfolds were all lower and HDL cholesterol higher among children who reported eating breakfast every day and showed evidence of graded and statistically significant associations across the breakfast frequency groups. However, no marked differences in adjusted mean values were observed for total and LDL cholesterol and diastolic blood pressure. Additional adjustment for socioeconomic status did not materially alter the results (Table S2); adjustment for physical activity in a subset of 1,581 children with objectively measured physical activity data had no effect on the results (Table S3; mean physical activity levels for each breakfast frequency group are also presented). To examine the extent to which these differences in risk markers were mediated by the association between breakfast consumption and adiposity, these analyses were repeated with additional adjustment for fat mass index and sum of skinfolds (Table 3). The differences in insulin resistance, HbA1c, glucose, and urate were still present, though smaller; differences in HDL cholesterol, triglycerides, C-reactive protein, and systolic blood pressure were greatly attenuated and no longer statistically significant. The associations between breakfast frequency and risk markers for type 2 diabetes and cardiovascular disease were consistent in the different ethnic groups, with no evidence of interaction with ethnicity.

**Table 2:** Means (geometric means) and differences (percent differences) in type 2 diabetes and cardiovascular risk markers by breakfast frequency in 4,116 study participants.

Risk Markers	Breakfast Frequency										*p* (Trend)
	Daily (*n* = 3,056)		Most Days (*n* = 450)		Some Days (*n* = 372)		Not Usually (*n* = 238)		Difference or Percent Difference(b)	
	Adjusted Mean	(95% CI)	Adjusted Mean	(95% CI)	Adjusted Mean	(95% CI)	Adjusted Mean	(95% CI)	Not Usually− Daily	(95% CI)	
Fat mass index (kg/m^5^)^a^	2.00	(1.96–2.03)	2.11	(2.03–2.19)	2.14	(2.06–2.23)	2.26	(2.15–2.38)	13.26^b^	(7.71–19.11)	<0.0001
Sum of skinfolds (mm)^a^	39.85	(39.02–40.70)	43.78	(41.81–45.84)	44.13	(41.96–46.40)	48.90	(45.96–52.03)	22.70^b^	(15.16–30.73)	<0.0001
Leptin (ng/ml)^a^	8.68	(8.32–9.05)	9.79	(8.97–10.68)	10.22	(9.29–11.24)	11.77	(10.46–13.23)	35.54^b^	(20.28–52.73)	<0.0001
Insulin (mmol/l)^a^	7.05	(6.82–7.30)	8.07	(7.58–8.58)	8.30	(7.76–8.88)	8.92	(8.22–9.67)	26.39^b^	(16.62–36.99)	<0.0001
Insulin resistance (HOMA)^a^	0.89	(0.86–0.92)	1.02	(0.96–1.08)	1.05	(0.98–1.12)	1.12	(1.04–1.22)	26.68^b^	(16.97–37.20)	<0.0001
HbA1c (%)^a^	5.23	(5.21–5.24)	5.23	(5.20–5.26)	5.26	(5.23–5.30)	5.29	(5.25–5.33)	1.21^b^	(0.38–2.04)	0.001
Glucose (mmol/l)^a^	4.50	(4.48–4.52)	4.55	(4.52–4.59)	4.59	(4.55–4.63)	4.54	(4.50–4.59)	0.97^b^	(−0.04 to 1.99)	<0.0001
C-reactive protein (mg/l)^a^	0.47	(0.45–0.50)	0.56	(0.50–0.64)	0.55	(0.48–0.63)	0.68	(0.57–0.80)	42.43^b^	(19.92–69.17)	<0.0001
Urate (mmol/l)^a^	0.22	(0.21–0.22)	0.22	(0.21–0.23)	0.22	(0.22–0.23)	0.23	(0.22–0.24)	6.38^b^	(2.93–9.94)	<0.0001
Triglycerides (mmol/l)^a^	0.79	(0.78–0.81)	0.81	(0.78–0.84)	0.81	(0.78–0.84)	0.84	(0.80–0.88)	5.92^b^	(0.76–11.34)	0.01
Total cholesterol (mmol/l)	4.57	(4.54–4.60)	4.53	(4.46–4.61)	4.52	(4.44–4.60)	4.60	(4.50–4.70)	0.04	(−0.06 to 0.14)	0.80
LDL cholesterol (mmol/l)	2.69	(2.66–2.72)	2.67	(2.61–2.73)	2.67	(2.60–2.74)	2.73	(2.64–2.81)	0.04	(−0.05 to 0.13)	0.78
HDL cholesterol (mmol/l)	1.53	(1.51–1.54)	1.50	(1.47–1.53)	1.48	(1.45–1.52)	1.49	(1.45–1.53)	−0.03	(−0.07 to 0.01)	0.003
Systolic BP (mmHg)	104.5	(104.0–105.0)	105.5	(104.5–106.5)	105.7	(104.6–106.8)	105.4	(104.0–106.7)	0.86	(−0.51 to 2.24)	0.02
Diastolic BP (mmHg)	62.7	(62.3–63.2)	62.9	(62.0–63.8)	63.2	(62.2–64.2)	63.2	(62.0–64.4)	0.45	(−0.77 to 1.66)	0.28

Adjusted means (or geometric means for those with footnote ^a^) from a multilevel model adjusted for age quartiles, month, ethnicity, and sex fitted as fixed effects and school fitted as a random effect. *p* (Trend) summarizes evidence for an ordered trend across the four breakfast frequency groups.

a Log transformed variables. Geometric means are given for these variables; percent differences are given between children who reported not usually eating breakfast and children who reported eating breakfast daily.

b Percent difference shown.

BP, blood pressure.

**Table 3:** Means (geometric means) and differences (percent differences) in type 2 diabetes and cardiovascular risk markers by breakfast frequency in 4,116 study participants: adjusted for adiposity markers.

Risk Markers	Breakfast Frequency										*p* (Trend)
	Daily (*n* = 3,056)		Most Days (*n* = 450)		Some Days (*n* = 372)		Not Usually (*n* = 238)		Difference or Percent Difference(b)		
	Adjusted Mean	(95% CI)	Adjusted Mean	(95% CI)	Adjusted Mean	(95% CI)	Adjusted Mean	(95% CI)	Not usually− Daily	(95% CI)	
Insulin (mmol/l)^a^	7.18	(6.95–7.89)	7.89	(7.45–8.36)	7.98	(7.50–8.49)	8.27	(7.67–8.91)	15.19^b^	(7.00–24.01)	<0.0001
Insulin resistance (HOMA)^a^	0.90	(0.87–0.99)	0.99	(0.94–1.05)	1.01	(0.95–1.07)	1.04	(0.97–1.12)	15.66^b^	(7.48–24.46)	<0.0001
HbA1c (%)^a^	5.23	(5.21–5.23)	5.23	(5.20–5.26)	5.26	(5.22–5.29)	5.28	(5.24–5.32)	0.97^b^	(0.15–1.80)	0.01
Glucose (mmol/l)^a^	4.50	(4.48–4.55)	4.55	(4.51–4.59)	4.59	(4.55–4.63)	4.54	(4.49–4.59)	0.84^b^	(−0.17 to 1.85)	<0.0001
C-reactive protein (mg/l)^a^	0.49	(0.47–0.53)	0.53	(0.48–0.59)	0.50	(0.44–0.56)	0.55	(0.48–0.64)	12.27^b^	(−3.40 to 30.48)	0.16
Urate (mmol/l)^a^	0.217	(0.21–0.22)	0.218	(0.21–0.22)	0.220	(0.21–0.23)	0.224	(0.22–0.23)	3.52^b^	(0.30–6.84)	0.02
Triglycerides (mmol/l)^a^	0.80	(0.78–0.80)	0.80	(0.77–0.83)	0.80	(0.77–0.83)	0.81	(0.77–0.85)	1.68^b^	(−3.09 to 6.68)	0.64
Total cholesterol (mmol/l)	4.57	(4.54–4.53)	4.53	(4.46–4.60)	4.51	(4.43–4.59)	4.59	(4.49–4.69)	0.02	(−0.08 to 0.12)	0.50
LDL cholesterol (mmol/l)	2.69	(2.66–2.66)	2.66	(2.60–2.73)	2.66	(2.59–2.73)	2.70	(2.62–2.79)	0.01	(−0.08 to 0.10)	0.58
HDL cholesterol (mmol/l)	1.52	(1.51–1.51)	1.51	(1.48–1.53)	1.50	(1.47–1.53)	1.52	(1.48–1.55)	−0.01	(−0.05 to 0.03)	0.18
Systolic BP (mmHg)	104.6	(104.1–105.3)	105.3	(104.3–106.3)	105.4	(104.3–106.5)	104.7	(103.4–106.1)	0.13	(−1.22 to 1.48)	0.25
Diastolic BP (mmHg)	62.9	(62.4–62.8)	62.8	(61.8–63.7)	62.9	(61.9–63.9)	62.7	(61.5–63.9)	−0.18	(−1.38 to 1.02)	0.87

Adjusted means are the predicted means (or geometric means for those with footnote ^a^) from a multilevel model adjusted for age quartiles, month, ethnicity, and sex fitted as fixed effects and school fitted as a random effect. *p* (Trend) summarizes evidence for an ordered trend across the four breakfast frequency groups.

a Log transformed variables; geometric means are given for these variables. Percentage differences are given between children who reported not usually eating breakfast and children who reported eating breakfast daily.

b Percent difference shown.

BP, blood pressure.

### Breakfast Content and Risk Markers for Type 2 Diabetes and Cardiovascular Disease: The Role of Energy and Nutrient Intakes

The associations between breakfast content and risk markers were examined among children who provided a detailed 24-hour dietary recall in the final 85 schools visited (Table 4). Fasting insulin levels and insulin resistance were appreciably lower among children eating high fibre cereal compared with children eating low fibre cereal, bread-based breakfast, biscuits, or other breakfast categories. As in the earlier analyses, particularly high insulin and HOMA-IR levels were observed in children not eating breakfast. The differences in insulin resistance remained after additional adjustment for adiposity was made. However, blood lipids and blood pressure showed no difference between the breakfast content groups.

**Table 4:** Means (geometric means) in type 2 diabetes and cardiovascular risk markers by breakfast type in 2,004 study participants.

Risk Markers	No Breakfast (*n* = 215)		High Fibre Cereal (*n* = 203)		Low Fibre Cereal (*n* = 808)		Bread/Toast Only (*n* = 265)		Biscuit Only (*n* = 68)		Other (*n* = 445)		*p*-Value*	*p*-Value**
	Adjusted Mean	(95% CI)	Adjusted Mean	(95% CI)	Adjusted Mean	(95% CI)	Adjusted Mean	(95% CI)	Adjusted Mean	(95% CI)	Adjusted Mean	(95% CI)		
Fat mass index (kg/m^5^)^a^	2.19	(2.09–2.30)	2.01	(1.92–2.12)	2.05	(2.00–2.10)	2.04	(1.95–2.13)	2.07	(1.90–2.26)	2.17	(2.10–2.25)	0.03	—
Sum of skinfolds (mm)^a^	45.14	(42.34–48.12)	38.71	(36.23–41.37)	40.58	(39.23–41.98)	40.44	(38.16–42.86)	40.71	(36.33–45.62)	43.12	(41.23–45.10)	0.08	—
Leptin (ng/ml)^a^	10.61	(9.33–12.07)	8.60	(7.53–9.82)	9.03	(8.38–9.73)	8.48	(7.55–9.54)	9.13	(7.32–11.38)	9.64	(8.78–10.59)	0.36	—
Insulin (mmol/l)^a^	7.64	(6.99–8.35)	6.28	(5.73–6.88)	7.24	(6.85–7.66)	7.02	(6.47–7.61)	7.34	(6.33–8.51)	7.63	(7.14–8.15)	0.004	0.02
Insulin resistance (HOMA)^a^	0.96	(0.88–1.05)	0.79	(0.72–0.86)	0.91	(0.86–0.96)	0.87	(0.81–0.95)	0.92	(0.80–1.07)	0.95	(0.89–1.02)	0.005	0.03
HbA1c (%)^a^	5.25	(5.21–5.29)	5.25	(5.20–5.29)	5.27	(5.24–5.29)	5.28	(5.24–5.32)	5.22	(5.14–5.30)	5.27	(5.23–5.30)	0.64	0.62
Glucose (mmol/l)^a^	4.51	(4.46–4.55)	4.44	(4.40–4.49)	4.47	(4.44–4.49)	4.45	(4.41–4.49)	4.45	(4.38–4.53)	4.47	(4.44–4.50)	0.79	0.86
C-reactive protein (mg/l)^a^	0.59	(0.50–0.70)	0.45	(0.37–0.53)	0.51	(0.47–0.56)	0.52	(0.45–0.61)	0.44	(0.33–0.60)	0.53	(0.47–0.60)	0.49	0.51
Urate (mmol/l)^a^	0.22	(0.22–0.23)	0.22	(0.21–0.23)	0.22	(0.22–0.22)	0.22	(0.22–0.23)	0.22	(0.21–0.23)	0.22	(0.22–0.23)	0.83	0.50
Triglycerides (mmol/l)^a^	0.83	(0.79–0.87)	0.83	(0.78–0.87)	0.83	(0.81–0.86)	0.82	(0.78–0.86)	0.81	(0.74–0.89)	0.84	(0.81–0.87)	0.92	0.97
Total cholesterol (mmol/l)	4.46	(4.36–4.56)	4.54	(4.44–4.64)	4.50	(4.44–4.55)	4.43	(4.34–4.51)	4.45	(4.28–4.62)	4.55	(4.48–4.62)	0.26	0.32
LDL cholesterol (mmol/l)	2.64	(2.55–2.72)	2.68	(2.59–2.77)	2.65	(2.60–2.70)	2.63	(2.56–2.71)	2.61	(2.46–2.76)	2.69	(2.63–2.75)	0.68	0.82
HDL cholesterol (mmol/l)	1.52	(1.47–1.56)	1.55	(1.50–1.59)	1.53	(1.51–1.55)	1.49	(1.45–1.52)	1.55	(1.47–1.62)	1.55	(1.52–1.58)	0.08	0.02
Systolic BP (mmHg)	104.0	(102.5–105.5)	104.3	(102.7–105.8)	104.4	(103.4–105.3)	104.2	(102.8–105.6)	103.9	(101.3–106.4)	104.4	(103.3–105.5)	0.99	0.96
Diastolic BP (mmHg)	62.2	(60.9–63.5)	62.9	(61.6–64.2)	63.0	(62.3–63.8)	62.6	(61.5–63.8)	62.5	(60.3–64.7)	62.2	(61.3–63.1)	0.62	0.30

Adjusted means are the predicted means (or geometric means for those with footnote ^a^) from a multilevel model with age quartiles, month, ethnicity, and sex as fixed effects and school as a random effect. Both *p*-values test for heterogeneity between types of breakfast and exclude children who did not have breakfast.

a log transformed variables; geometric means and interquartile ranges are given for these variables.

*p-Values are adjusted for age in quartiles, month, ethnicity, sex, and school (random effect).

**p-Values are adjusted for age in quartiles, month, ethnicity, sex, school (random effect) and also for adiposity (represented by fat mass index).

BP, blood pressure.

In order to examine whether the association between breakfast frequency and type 2 diabetes risk markers reflected differences in energy or nutrient intakes, the associations between breakfast frequency and total energy, energy density, and nutrient intakes were analysed for the children with 24-hour recall data (Table S4). Children who did not eat breakfast every day had lower total energy intakes but higher energy density and percentages of energy from fat (particularly monounsaturated fat). They also had lower intakes of carbohydrates and total non-starch polysaccharides. Their micronutrient intakes were all also markedly lower including vitamin B12, folate, vitamin C, calcium, and iron. In order to determine whether these differences specifically applied to breakfast consumption or were part of a general dietary pattern, these analyses were repeated after omitting all food or drinks consumed between 6 am and 9 am (Table S5). The intakes of total energy and macronutrients at other times of day did not differ markedly between the breakfast frequency groups, but dietary energy density and intakes of micronutrients—particularly folate, vitamin C, and calcium—were still appreciably lower at other times of the day in children not eating breakfast. The extent to which differences in energy and nutrient intakes between breakfast frequency groups could account for the associations between breakfast frequency and type 2 diabetes risk markers were examined (Table S6). Individual adjustments for total energy, energy density, macronutrients (total fat, carbohydrates, and protein), non-starch polysaccharides, and micronutrients (vitamin B12, folate, vitamin C, calcium, and iron) did not materially affect the type 2 diabetes risk marker differences between children who did or did not eat breakfast daily.

## Discussion

In the present study, children who reported not eating breakfast every day had higher levels of risk markers for type 2 diabetes and cardiovascular disease than children who ate breakfast every day. These associations were not confounded by socioeconomic status and physical activity. The higher insulin resistance, HbA1c, and fasting glucose levels in children who did not eat breakfast every day remained after adjustment for adiposity. Among children who ate breakfast daily, children who ate high fibre cereals had lower insulin resistance compared to children who ate breakfast with low fibre cereal or other content. Children who did not eat breakfast every day had lower total energy intakes but more energy dense diets with higher fat intakes as a percentage of energy, and lower fibre and micronutrient intakes. These differences were not completely explained by the lack of the breakfast meal as differences in energy density and micronutrients persisted throughout the rest of the day. However, these differences in diet did not explain the differences in risk markers (particularly in type 2 diabetes risk markers) between breakfast eaters and non-breakfast eaters.

### Comparison with Previous Studies

In the current study about a quarter of children reported not eating breakfast daily. This proportion is lower than that in a study in UK adolescents from different ethnic groups, in which over 40% did not eat breakfast daily [14]; this probably reflects the increase in breakfast skipping with increasing age [28]. Most of the previous studies that have examined the association between breakfast consumption and risk markers for type 2 diabetes and cardiovascular disease have been in adults. Longitudinal studies have reported associations between breakfast skipping and an increased risk of type 2 diabetes [6] and with insulin resistance and glycaemia, even after adjustment for adiposity [29]. Other studies in adults have suggested that skipping breakfast is associated with increased adiposity, adverse blood pressure, and blood lipid profiles [8] and with cardiovascular disease [30]. However, few trials of breakfast interventions have so far been conducted; a small randomised controlled trial in normal weight Finnish adults showed a reduction in total serum cholesterol in individuals given breakfast compared to a control group [31]. The current finding that the high fibre breakfast cereal group have the lowest insulin resistance is consistent with previous studies in adults and reflects the importance of the type of breakfast consumed on disease risk [32].

In children, several studies have reported associations between skipping breakfast and increased adiposity, consistent with our findings [33],[34]. Longitudinal data also show associations between breakfast skipping and weight gain [35]. In a large cross-sectional study of over 3,500 adolescents (the HELENA study), breakfast skipping was positively associated with insulin resistance in males but not in females [36]. However, to the best of our knowledge no studies have reported an additional effect of breakfast type and cardiometabolic risk in children.

Studies in both adults and children have shown that individuals who do not eat breakfast every day have poorer overall diet quality, with higher fat intakes and lower intakes of carbohydrates, fibre, and micronutrients, particularly vitamin C, calcium, and iron [3]. This is consistent with food group analyses in both adolescents and young children; a previous study in older children reported that children who skipped breakfast had a lower mean number of servings of fruits, vegetables, grain products, and milk products [14]. The finding that children who ate breakfast regularly had higher energy intakes is consistent with some adult studies [37] and may suggest that eating breakfast, despite increasing energy intake, has independent beneficial metabolic effects. However, the results are paradoxical as the group eating breakfast regularly also had lower levels of adiposity and risk markers for type 2 diabetes (and no difference in physical activity levels). This paradox could potentially be explained by selective under-reporting, with children who do not eat breakfast systematically under-reporting energy intake from other meals and snacks through the rest of the day. Under-reporting of energy intake is a common phenomenon in dietary assessment studies, and has already been reported in this study population [38]. Further studies are needed to resolve this issue.

### Strengths and Limitations

The current study benefits from a large sample size, drawn from a multi-ethnic population of school children based in three UK cities. Although participation rates were moderate, with some evidence of under-representation of boys, black African Caribbeans, and economically inactive families, this would not be expected to invalidate the associations reported in this paper. The sampling strategy, which ensured the inclusion of balanced numbers of white European, South Asian, and black African Caribbean children, allowed associations between breakfast patterns and risk markers to be reported in a way that took account for differences between ethnic groups and without biasing the observed associations. Although usual breakfast frequency was self-reported by the children, there was close agreement with the reported breakfast consumption in the 24-hour recall that was conducted by a trained nutritionist. Among children who reported eating breakfast daily, most days, some days, and not usually, the proportions with a breakfast meal documented in their 24-hour recall were 94%, 85%, 61%, and 30%, respectively. Dietary data were collected using a single 24-hour recall, which provides an unbiased though imprecise estimate of usual nutrient intakes [39]. We have already reported elsewhere [40] that our nutrient intake data were very consistent with those of children of a similar age-group in the 2000 National Diet and Nutrition Survey, in which prospective, 7-day, weighed food diaries were used to assess food intakes [41] and showed expected associations between estimated fat intakes and blood lipids [42]. Assessment of body fat was primarily based on fat mass index derived from bioelectrical impedance, a more valid indicator of body fat than BMI in this multi-ethnic population [19]. The cross-sectional nature of the study is particularly appropriate for documenting short-term associations between eating patterns and emerging diabetes risk, though it cannot determine the chronological sequence of associations observed and therefore establish the direction of causality. Although the study data were collected a few years ago (2004–2007), patterns of breakfast consumption and breakfast cereal content have not changed appreciably since that time, suggesting that the observed associations between breakfast consumption and type 2 diabetes risk markers remain highly relevant.

### Implications

The observed associations suggest that regular breakfast consumption, particularly involving consumption of a high fibre cereal, could protect against the early development of type 2 diabetes risk, partly though not entirely through effects on adiposity levels. The findings from the present study suggest that this association is independent of measured confounding factors and potentially causal, though experimental evidence is now needed to make causal inferences. In particular, randomized controlled trials examining the effect of providing a high fibre cereal breakfast, carried out in children either not currently eating breakfast or eating a low fibre breakfast, would be particularly informative. However, even if the association between breakfast consumption and type 2 diabetes risk is causal, the mechanism of the observed associations remains uncertain. One important possibility is that these results directly reflect the effects of breakfast consumption on specific nutrients. Differences in fibre intake between the groups are an important possibility, in light of the evidence from observational studies both in adults and children that show that high intakes of fibre, particularly cereal fibre, are associated with lower risks of type 2 diabetes and insulin resistance [43]. However, other differences in dietary intakes could also be important, for example whole grain intakes [9]. Alternatively, the observations could reflect the importance of increased meal frequency, or the timings and distribution of energy intakes over the day [6]. Further studies will be needed to resolve which of these potential explanations is most important.

The findings reported here are important given the high prevalence of overweight/obesity in children from the UK and other Western countries, the increasing prevalence of type 2 diabetes, and the evidence from this study and others that substantial proportions of children do not eat breakfast daily [14] and consume less dietary fibre than recommended [28],[44]. Data from this study suggest that encouraging all children who do not eat breakfast daily to do so might reduce population-wide fasting insulin levels by ∼4%, while encouraging all children who currently eat a low fibre breakfast to instead consume a high fibre type might reduce population-wide fasting insulin levels by a larger amount (∼11%–12%). Further experimental studies exploring the effect of providing breakfast to children not currently consuming it, the effect of changing from a low to a high fibre breakfast, and the acceptability of these interventions to children and their families are therefore important priorities for further research.

### Conclusions

Children who ate breakfast regularly, particularly a high fibre cereal breakfast, had a more favourable type 2 diabetes risk profile, particularly lower levels of insulin resistance. These differences were independent of adiposity and potential confounders, particularly socioeconomic position and physical activity. Experimental studies are needed to establish whether modification of breakfast consumption patterns can reduce emerging type 2 diabetes risk.

## Supporting Information

Table S1 **Population characteristics and risk markers: comparisons of children included and excluded from analyses.**
(DOCX)

Table S2 **Risk markers by breakfast consumption in all participants: additional adjustment for socio-economic status.**
(DOCX)

Table S3 **Risk markers by breakfast frequency in all participants: adjusted for physical activity.**
(DOCX)

Table S4 **24 hour energy and nutrient intake by frequency of breakfast consumption.**
(DOCX)

Table S5 **Energy and nutrient intakes calculated for the rest of the day (excluding breakfast slot) by frequency of breakfast.**
(DOCX)

Table S6 **The difference in risk markers between children who do and do not eat breakfast daily.**
(DOCX)

Table S7 **Raw data for variables indicated in Table 1.**
(DOCX)

Checklist S1 **STROBE Statement – checklist of items to be included in reports on cross sectional studies.**
(DOC)

## Abbreviations

HbA1c: glycated haemoglobin
HDL: high density lipoprotein
HOMA: homeostasis model assessment
LDL: low density lipoprotein
