# Supplementary material for: Regular Breakfast Consumption and Type 2 Diabetes Risk Markers in 9- to 10-Year-Old Children in the Child Heart and Health Study in England (CHASE): A Cross-Sectional Analysis
Source: PLoS Med. 2014 Sep 2;11(9):e1001703. doi: 10.1371/journal.pmed.1001703 (PMC4151989; doi:10.1371/journal.pmed.1001703)
Supplement: Table S1 — Population characteristics and risk markers: comparisons of children included and excluded from analyses. (DOCX) [file pmed.1001703.s001.docx]

**Table S1:** Sociodemographic characteristics and risk markers for type 2 diabetes and cardiovascular disease: comparisons of children included and excluded from analyses

|  | |  | | Included children (n=4116) | | Excluded children (n=1771) | | p (no difference) |
| --- | --- | --- | --- | --- | --- | --- | --- | --- |
| Socio-demographic characteristics | | | |  |  |  |  |  |
| Sex (% female) | | | | 53 | | 47 | | 0.001 |
| Ethnic group | | | |  | |  | |  |
|  | % white European | | | 24 | | 23 | |  |
|  | % black African Caribbean | | | 26 | | 31 | |  |
|  | % South Asian | | | 27 | | 26 | | 0.001 |
| Socio-economic status ¹ | | | |  | |  | |  |
|  | % Managerial/professional | | | 28 | | 28 | |  |
|  | % Intermediate | | | 26 | | 24 | |  |
|  | % Routine & manual | | | 30 | | 29 | |  |
|  | % Economically inactive | | | 10 | | 13 | | 0.002 |
| Age, years (95% reference range) | | | | 10.0 (9.3 - 10.6) | | 9.9 (9.3 - 10.6) | | 0.99 |
|  | |  | | Included children (n=4116) | | Excluded children (n=496) | |  |
|  | |  | |  |  |  |  |  |
| Risk markers for type 2 diabetes and cardiovascular disease | | | | Adjusted mean |  | Adjusted mean |  |  |
|  |  |  |  |  | (95% CI) |  | (95% CI) |  |
| Fat mass index (kg/m5) ² | | |  | 2.04 | (2.00, 2.07) | 2.06 | (1.98, 2.13) | 0.59 |
| Sum of skinfolds (mm)² | | |  | 41.08 | (40.31, 41.86) | 41.11 | (39.26, 43.05) | 0.98 |
| Leptin (ng/mL) ² | | |  | 9.05 | (8.72, 9.40) | 9.54 | (8.73, 10.42) | 0.25 |
| Insulin (mmol/L) ² | | |  | 7.36 | (7.13, 7.59) | 7.29 | (6.84, 7.77) | 0.76 |
| Insulin resistance (HOMA)² | | |  | 0.93 | (0.90, 0.96) | 0.92 | (0.87, 0.98) | 0.91 |
| HbA1c (%) ² | | |  | 5.23 | (5.22, 5.25) | 5.26 | (5.23, 5.29) | 0.10 |
| Glucose (mmol/L) ² | | |  | 4.52 | (4.50, 4.54) | 4.55 | (4.52, 4.59) | 0.06 |
| C-reactive protein (mg/L) ² | | |  | 0.50 | (0.48, 0.52) | 0.54 | (0.48, 0.61) | 0.19 |
| Urate (mmol/L) ² | | |  | 0.22 | (0.22, 0.22) | 0.22 | (0.21, 0.22) | 0.45 |
| Triglycerides (mmol/L) ² | | |  | 0.80 | (0.79, 0.81) | 0.83 | (0.80, 0.86) | 0.06 |
| Total cholesterol (mmol/L) | | |  | 4.56 | (4.53, 4.59) | 4.59 | (4.52, 4.67) | 0.40 |
| LDL- cholesterol (mmol/L) | | |  | 2.69 | (2.67, 2.72) | 2.73 | (2.67, 2.80) | 0.21 |
| HDL- cholesterol (mmol/L) | | |  | 1.52 | (1.50, 1.53) | 1.51 | (1.49, 1.54) | 0.94 |
| Systolic BP (mmHg) | | |  | 104.8 | (104.3, 105.3) | 104.4 | (103.3, 105.4) | 0.41 |
| Diastolic BP (mmHg) | | |  | 62.8 | (62.4, 63.3) | 63.5 | (62.5, 64.4) | 0.20 |

Data are presented for all 4116 children included in the analyses. Sociodemographic comparisons are based on 1771 children with sociodemographic information but missing data on other key variables; risk marker comparisons are based on 496 children with complete data on risk markers but missing data on other key variables.

All adjusted means are adjusted for sex, age (in quartiles), month, ethnicity and school (random effect).

¹ 351 children had missing or unclassified data on parental occupation and therefore were not included in an NS-SEC category.

² Geometric means are given for log transformed variables.
