# Supplementary material for: Regular Breakfast Consumption and Type 2 Diabetes Risk Markers in 9- to 10-Year-Old Children in the Child Heart and Health Study in England (CHASE): A Cross-Sectional Analysis
Source: PLoS Med. 2014 Sep 2;11(9):e1001703. doi: 10.1371/journal.pmed.1001703 (PMC4151989; doi:10.1371/journal.pmed.1001703)
Supplement: Table S2 — Risk markers by breakfast consumption in all participants: additional adjustment for socio-economic status. (DOCX) [file pmed.1001703.s002.docx]

**Table S2:** Means and Geometric means of type 2 diabetes and cardiovascular disease risk markers by breakfast consumption in all participants: additional adjustment for socio-economic status

|  | Daily breakfast  (n=2914) | | Most days  (n=416) | | Some days  (n=340) | | No, not usually  (n=216) | | p (trend) |
| --- | --- | --- | --- | --- | --- | --- | --- | --- | --- |
|  | Mean | (95% CI) | Mean | (95% CI) | Mean | (95% CI) | Mean | (95% CI) |  |
| Fat mass index (kg/m5) ¹ | 2.01 | (1.97, 2.04) | 2.13 | (2.05, 2.21) | 2.15 | (2.07, 2.24) | 2.28 | (2.16, 2.40) | <0.0001 |
| Sum of skinfolds (mm) ¹ | 40.09 | (39.25, 40.95) | 44.38 | (42.31, 46.55) | 44.26 | (42.00, 46.64) | 48.82 | (45.74, 52.10) | <0.0001 |
| Leptin (ng/mL) ¹ | 8.75 | (8.39, 9.13) | 10.03 | (9.16, 10.99) | 10.19 | (9.23, 11.26) | 11.74 | (10.38, 13.28) | <0.0001 |
| Insulin (mmol/L) ¹ | 7.05 | (6.82, 7.29) | 8.16 | (7.65, 8.69) | 8.30 | (7.74, 8.89) | 8.83 | (8.11, 9.61) | <0.0001 |
| Insulin resistance (HOMA)¹ | 0.89 | (0.86, 0.92) | 1.03 | (0.97, 1.09) | 1.05 | (0.98, 1.12) | 1.12 | (1.03, 1.21) | <0.0001 |
| HbA1c (%) ¹ | 5.23 | (5.21, 5.24) | 5.24 | (5.20, 5.27) | 5.27 | (5.23, 5.31) | 5.29 | (5.24, 5.33) | 0.001 |
| Glucose (mmol/L) ¹ | 4.50 | (4.48, 4.52) | 4.56 | (4.52, 4.59) | 4.60 | (4.56, 4.64) | 4.55 | (4.51, 4.60) | <0.0001 |
| C-reactive protein (mg/L) ¹ | 0.48 | (0.45, 0.50) | 0.58 | (0.51, 0.66) | 0.56 | (0.48, 0.64) | 0.69 | (0.58, 0.83) | <0.0001 |
| Urate (mmol/L) ¹ | 0.22 | (0.21, 0.22) | 0.22 | (0.22, 0.23) | 0.22 | (0.22, 0.23) | 0.23 | (0.22, 0.24) | <0.0001 |
| Triglycerides (mmol/L) ¹ | 0.80 | (0.78, 0.81) | 0.81 | (0.78, 0.85) | 0.80 | (0.77, 0.84) | 0.84 | (0.80, 0.89) | 0.05 |
| Total cholesterol (mmol/L) | 4.57 | (4.54, 4.60) | 4.54 | (4.46, 4.61) | 4.52 | (4.44, 4.61) | 4.63 | (4.52, 4.73) | 0.97 |
| LDL- cholesterol (mmol/L) | 2.69 | (2.66, 2.72) | 2.68 | (2.61, 2.74) | 2.68 | (2.61, 2.75) | 2.75 | (2.66, 2.84) | 0.56 |
| HDL- cholesterol (mmol/L) | 1.53 | (1.51, 1.54) | 1.50 | (1.47, 1.53) | 1.49 | (1.45, 1.52) | 1.50 | (1.46, 1.54) | 0.01 |
| Systolic BP (mmHg) | 104.5 | (103.9, 105.0) | 105.6 | (104.6, 106.7) | 105.6 | (104.5, 106.8) | 105.5 | (104.0, 106.9) | 0.01 |
| Diastolic BP (mmHg) | 62.7 | (62.2, 63.2) | 62.9 | (62.0, 63.9) | 63.0 | (62.0, 64.0) | 63.4 | (62.2, 64.7) | 0.26 |

Abbreviations: BP -blood pressure; CI -confidence intervals. HDL high-density lipoprotein, HOMA homeostasis model assessment, HbA1c glycated haemoglobin, LDL low density lipoprotein.

¹ log transformed variables; geometric means and interquartile ranges are given for these variables.

Means/geometric means ¹ are adjusted for socio-economic status, age in quartiles, month, ethnicity, sex and school (random effect).

Analyses are based on all 4116 children, but excluding 230 children with missing data on NS-SEC.
