# Supplementary material for: Regular Breakfast Consumption and Type 2 Diabetes Risk Markers in 9- to 10-Year-Old Children in the Child Heart and Health Study in England (CHASE): A Cross-Sectional Analysis
Source: PLoS Med. 2014 Sep 2;11(9):e1001703. doi: 10.1371/journal.pmed.1001703 (PMC4151989; doi:10.1371/journal.pmed.1001703)
Supplement: Table S3 — Risk markers by breakfast frequency in all participants: adjusted for physical activity. (DOCX) [file pmed.1001703.s003.docx]

**Table S3:** Means and geometric means of type 2 diabetes and cardiovascular disease risk markers by breakfast frequency in all participants: adjusted for physical activity

|  | Daily breakfast (n=1295) | | Most days  (n=121) | | Some days  (n=107) | | No, not usually  (n=58) | | p (trend) |
| --- | --- | --- | --- | --- | --- | --- | --- | --- | --- |
|  | Mean | (95% CI) | Mean | (95% CI) | Mean | (95% CI) | Mean | (95% CI) |  |
| Physical activity (counts per min) | 481 | (473, 489) | 495 | (478, 512) | 477 | (459, 495) | 488 | (464, 513) | 0.57 |
| Fat mass index (kg/m5) ¹ | 2.05 | (2.01, 2.09) | 2.17 | (2.04, 2.31) | 2.21 | (2.07, 2.36) | 2.41 | (2.20, 2.64) | <0.0001 |
| Sum of skinfolds (mm) ¹ | 40.19 | (39.16, 41.26) | 43.62 | (40.13, 47.41) | 45.19 | (41.37, 49.37) | 48.19 | (42.72, 54.36) | <0.0001 |
| Leptin (ng/mL) ¹ | 9.06 | (8.56, 9.60) | 9.66 | (8.23, 11.33) | 11.23 | (9.48, 13.30) | 11.20 | (8.91, 14.09) | 0.004 |
| Insulin (mmol/L) ¹ | 7.17 | (6.81, 7.55) | 7.36 | (6.59, 8.22) | 8.41 | (7.48, 9.46) | 8.69 | (7.44, 10.15) | <0.001 |
| Insulin resistance (HOMA)¹ | 0.90 | (0.85, 0.94) | 0.92 | (0.83, 1.03) | 1.06 | (0.94, 1.19) | 1.09 | (0.93, 1.27) | <0.001 |
| HbA1c (%) ¹ | 5.25 | (5.23, 5.27) | 5.23 | (5.17, 5.29) | 5.30 | (5.24, 5.37) | 5.30 | (5.22, 5.39) | 0.09 |
| Glucose (mmol/L) ¹ | 4.44 | (4.42, 4.46) | 4.46 | (4.40, 4.52) | 4.53 | (4.47, 4.60) | 4.43 | (4.35, 4.52) | 0.07 |
| C-reactive protein (mg/L) ¹ | 0.50 | (0.47, 0.54) | 0.62 | (0.50, 0.78) | 0.74 | (0.58, 0.94) | 0.82 | (0.59, 1.13) | <0.0001 |
| Urate (mmol/L) ¹ | 0.22 | (0.21, 0.22) | 0.22 | (0.21, 0.23) | 0.23 | (0.22, 0.24) | 0.23 | (0.22, 0.25) | 0.004 |
| Triglycerides (mmol/L) ¹ | 0.82 | (0.79, 0.84) | 0.86 | (0.81, 0.92) | 0.85 | (0.79, 0.91) | 0.87 | (0.79, 0.96) | 0.08 |
| Total cholesterol (mmol/L) | 4.51 | (4.46, 4.55) | 4.47 | (4.34, 4.60) | 4.44 | (4.30, 4.58) | 4.37 | (4.18, 4.56) | 0.112 |
| LDL- cholesterol (mmol/L) | 2.65 | (2.61, 2.69) | 2.60 | (2.49, 2.72) | 2.62 | (2.50, 2.75) | 2.54 | (2.37, 2.71) | 0.17 |
| HDL- cholesterol (mmol/L) | 1.5 | (1.5, 1.6) | 1.5 | (1.5, 1.6) | 1.5 | (1.4, 1.5) | 1.5 | (1.4, 1.5) | 0.02 |
| Systolic BP (mmHg) | 104.2 | (103.3, 105.0) | 104.4 | (102.5, 106.4) | 104.9 | (102.9, 107.0) | 104.0 | (101.3, 106.8) | 0.65 |
| Diastolic BP (mmHg) | 62.7 | (62.0, 63.4) | 62.0 | (60.3, 63.6) | 62.5 | (60.7, 64.3) | 62.5 | (60.0, 64.9) | 0.63 |

Abbreviations: BP -blood pressure; CI -confidence intervals. HDL high-density lipoprotein, HOMA homeostasis model assessment, HbA1c glycated haemoglobin, LDL low density lipoprotein.

¹ log transformed variables; geometric means and interquartile ranges are given for these variables.

Means/geometric means ¹ are adjusted for physical activity, age in quartiles, month, ethnicity, sex and school (random effect).

Analyses based on 1581 participants with objective physical activity measurements.
