# Supplementary material for: Regular Breakfast Consumption and Type 2 Diabetes Risk Markers in 9- to 10-Year-Old Children in the Child Heart and Health Study in England (CHASE): A Cross-Sectional Analysis
Source: PLoS Med. 2014 Sep 2;11(9):e1001703. doi: 10.1371/journal.pmed.1001703 (PMC4151989; doi:10.1371/journal.pmed.1001703)
Supplement: Table S4 — 24 hour energy and nutrient intake by frequency of breakfast consumption. (DOCX) [file pmed.1001703.s004.docx]

**Table S4:** 24 hour energy and nutrient intake by frequency of breakfast consumption: in 1899 children with a 24 hour recall and data on usual breakfast consumption

|  | Daily breakfast (n=1554) | | Most days (n=153) | | Some days (n=132) | | No, not usually (n=60) | | p (trend) |
| --- | --- | --- | --- | --- | --- | --- | --- | --- | --- |
|  | Mean | (95% CI) | Mean | (95% CI) | Mean | (95% CI) | Mean | (95% CI) |  |
| Energy (kcals) | 1,867 | (1,838, 1,897) | 1,820 | (1,742, 1,899) | 1,774 | (1,690, 1,858) | 1,567 | (1,444, 1,690) | <0.0001 |
| Energy density | 7.0 | (6.9, 7.1) | 7.1 | (6.8, 7.3) | 7.3 | (7.1, 7.6) | 7.4 | (7.0, 7.8) | 0.002 |
| Fat % energy | 34.1 | (33.8, 34.5) | 34.6 | (33.5, 35.6) | 35.3 | (34.2, 36.3) | 35.8 | (34.2, 37.4) | 0.01 |
| Saturated fat % energy | 12.7 | (12.5, 12.9) | 12.6 | (12.1, 13.1) | 12.6 | (12.1, 13.2) | 12.7 | (11.8, 13.6) | 0.85 |
| Monounsaturated fat % energy | 11.3 | (11.1, 11.5) | 11.5 | (11.0, 11.9) | 11.7 | (11.2, 12.2) | 12.5 | (11.8, 13.2) | <0.001 |
| Polyunsaturated fat % energy | 6.5 | (6.3, 6.7) | 6.8 | (6.3, 7.2) | 6.8 | (6.3, 7.3) | 6.7 | (6.0, 7.5) | 0.15 |
| Carbohydrate % energy | 52.2 | (51.9, 52.6) | 51.9 | (50.8, 52.9) | 51.1 | (49.9, 52.2) | 49.6 | (47.9, 51.3) | 0.001 |
| Sugars % energy | 22.6 | (22.2, 23.0) | 22.4 | (21.3, 23.5) | 22.5 | (21.3, 23.6) | 20.9 | (19.2, 22.7) | 0.13 |
| Starch, Dextrins % energy | 29.1 | (28.7, 29.5) | 29.0 | (28.1, 30.0) | 28.1 | (27.0, 29.1) | 27.9 | (26.4, 29.5) | 0.02 |
| Total NSP(g) | 11.9 | (11.6, 12.1) | 11.7 | (11.1, 12.4) | 11.7 | (11.0, 12.4) | 11.6 | (10.6, 12.7) | 0.44 |
| Protein % energy | 13.3 | (13.1, 13.5) | 13.3 | (12.8, 13.8) | 13.4 | (12.8, 13.9) | 14.3 | (13.4, 15.1) | 0.09 |
| Vitamin B12 (μg) ¹ | 3.0 | (2.9, 3.1) | 2.8 | (2.5, 3.1) | 2.7 | (2.4, 3.1) | 2.5 | (2.1, 3.0) | 0.01 |
| Folate (μg) ¹ | 207 | (202, 212) | 187 | (176, 199) | 166 | (156, 177) | 142 | (129, 155) | <0.0001 |
| Vitamin C (mg) ¹ | 84.0 | (80.2, 88.0) | 81.6 | (72.2, 92.2) | 67.0 | (58.7, 76.4) | 59.5 | (49.0, 72.2) | <0.0001 |
| Calcium (mg) ¹ | 735 | (720, 750) | 713 | (674, 754) | 633 | (596, 673) | 572 | (523, 625) | <0.0001 |
| Iron (mg) ¹ | 9.0 | (8.9, 9.2) | 8.3 | (7.9, 8.6) | 8.1 | (7.7, 8.5) | 7.6 | (7.1, 8.2) | <0.0001 |

Abbreviations: CI -confidence intervals, NSP non-starch polysaccharides.

¹ log transformed variables; geometric means and interquartile ranges are given for these variables.

Means and geometric means are adjusted for age in quartiles, day, month, ethnic subgroup, sex and school (random effect). All micronutrients and NSP are also adjusted for total energy.
