# Supplementary material for: Regular Breakfast Consumption and Type 2 Diabetes Risk Markers in 9- to 10-Year-Old Children in the Child Heart and Health Study in England (CHASE): A Cross-Sectional Analysis
Source: PLoS Med. 2014 Sep 2;11(9):e1001703. doi: 10.1371/journal.pmed.1001703 (PMC4151989; doi:10.1371/journal.pmed.1001703)
Supplement: Table S6 — The difference in risk markers between children who do and do not eat breakfast daily. (DOCX) [file pmed.1001703.s006.docx]

**Table S6:** The difference in type 2 diabetes risk markers between children who do and do not eat breakfast daily: in 1899 children with a 24 hour recall and data on usual breakfast consumption

|  | Adjustments | Percentage difference | (95% CI) | p (no difference) |
| --- | --- | --- | --- | --- |
| Insulin resistance ¹ | Standard ² | 15.2 | (7.3, 23.7) | <0.0001 |
| (HOMA) | Standard plus total energy | 16.8 | (8.8, 25.4) | <0.0001 |
|  | Standard plus energy density | 15.1 | (7.1, 23.5) | <0.001 |
|  | Standard plus macronutrients | 15.7 | (7.8, 24.2) | <0.0001 |
|  | Standard plus non-starch polysaccharides | 15.6 | (7.7, 24.1) | <0.0001 |
|  | Standard plus micronutrients | 16.9 | (8.7, 25.6) | <0.0001 |
| HbA1c (%) ¹ | Standard ² | 0.7 | (0.0, 1.5) | 0.05 |
|  | Standard plus total energy | 0.8 | (0.0, 1.5) | 0.04 |
|  | Standard plus energy density | 0.8 | (0.0, 1.5) | 0.05 |
|  | Standard plus macronutrients | 0.8 | (0.0, 1.5) | 0.05 |
|  | Standard plus non-starch polysaccharides | 0.8 | (0.0, 1.5) | 0.04 |
|  | Standard plus micronutrients | 0.9 | (0.1, 1.7) | 0.02 |
| Glucose (mmol/L) ¹ | Standard ² | 1.4 | (0.5, 2.2) | 0.001 |
|  | Standard plus total energy | 1.5 | (0.6, 2.3) | <0.001 |
|  | Standard plus energy density | 1.4 | (0.6, 2.2) | 0.001 |
|  | Standard plus macronutrients | 1.4 | (0.5, 2.2) | 0.001 |
|  | Standard plus non-starch polysaccharides | 1.4 | (0.6, 2.2) | 0.001 |
|  | Standard plus micronutrients | 1.5 | (0.6, 2.3) | <0.001 |

Abbreviations: CI confidence intervals, HOMA homeostasis model assessment, HbA1c glycated haemoglobin.

¹ log transformed variables; ² Standard adjustments are made for age in quartiles, month, ethnicity, sex and school (random effect).
