# Supplementary material for: Regular Breakfast Consumption and Type 2 Diabetes Risk Markers in 9- to 10-Year-Old Children in the Child Heart and Health Study in England (CHASE): A Cross-Sectional Analysis
Source: PLoS Med. 2014 Sep 2;11(9):e1001703. doi: 10.1371/journal.pmed.1001703 (PMC4151989; doi:10.1371/journal.pmed.1001703)
Supplement: Table S7 — Raw data for variables indicated in Table 1. (DOCX) [file pmed.1001703.s007.docx]

**Table S7. Raw data for variables indicated in Table 1.**

|  | **Breakfast frequency reported as** | | | | | | | | ***p*-Value** |
| --- | --- | --- | --- | --- | --- | --- | --- | --- | --- |
|  | **Every day** | | **Most days** | | **Some days** | | **Not usually** | |  |
| Fat mass index (kg/m^5^) | 2.15 | 0.83 | 2.28 | 0.94 | 2.34 | 0.96 | 2.46 | 1.01 | *<* 0.0001 |
| Sum of skinfolds (mm) | 44.87 | 22.99 | 49.62 | 24.75 | 50.16 | 26.06 | 55.88 | 27.47 | < 0.0001 |
| Leptin (ng/mL) | 13.46 | 14.03 | 14.7 | 13.62 | 16.31 | 16.48 | 18.37 | 15.8 | < 0.0001 |
| Insulin (mmol/L) | 8.59 | 7.13 | 9.93 | 8.07 | 10.35 | 7.47 | 10.7 | 6.51 | < 0.0001 |
| Insulin resistance (HOMA) | 1.07 | 0.85 | 1.24 | 0.97 | 1.3 | 0.92 | 1.34 | 0.79 | < 0.0001 |
| HbA1c (%) | 5.25 | 0.32 | 5.25 | 0.33 | 5.28 | 0.34 | 5.31 | 0.3 | 0.006 |
| Glucose (mmol/L) | 4.50 | 0.35 | 4.56 | 0.36 | 4.61 | 0.36 | 4.56 | 6.40 | < 0.0001 |
| C-reactive protein (mg/L) | 1.33 | 3.62 | 1.51 | 4.24 | 1.70 | 4.26 | 2.38 | 7.86 | 0.001 |
| Urate (mmol/L) | 0.22 | 0.05 | 0.23 | 0.06 | 0.23 | 0.05 | 0.23 | 0.05 | 0.009 |
| Triglycerides (mmol/L) | 0.87 | 0.40 | 0.87 | 0.40 | 0.88 | 0.38 | 0.90 | 0.41 | 0.70 |

Values shown are mean, SD.

*p*-Values test for unordered differences between breakfast groups and are based on ANOVA tests.
